# Supplementary figures and images for: Metabolic and Chaperone Gene Loss Marks the Origin of Animals: Evidence for Hsp104 and Hsp78 Chaperones Sharing Mitochondrial Enzymes as Clients
Source: PLoS One. 2015 Feb 24;10(2):e0117192. doi: 10.1371/journal.pone.0117192 (PMC4339202; doi:10.1371/journal.pone.0117192)

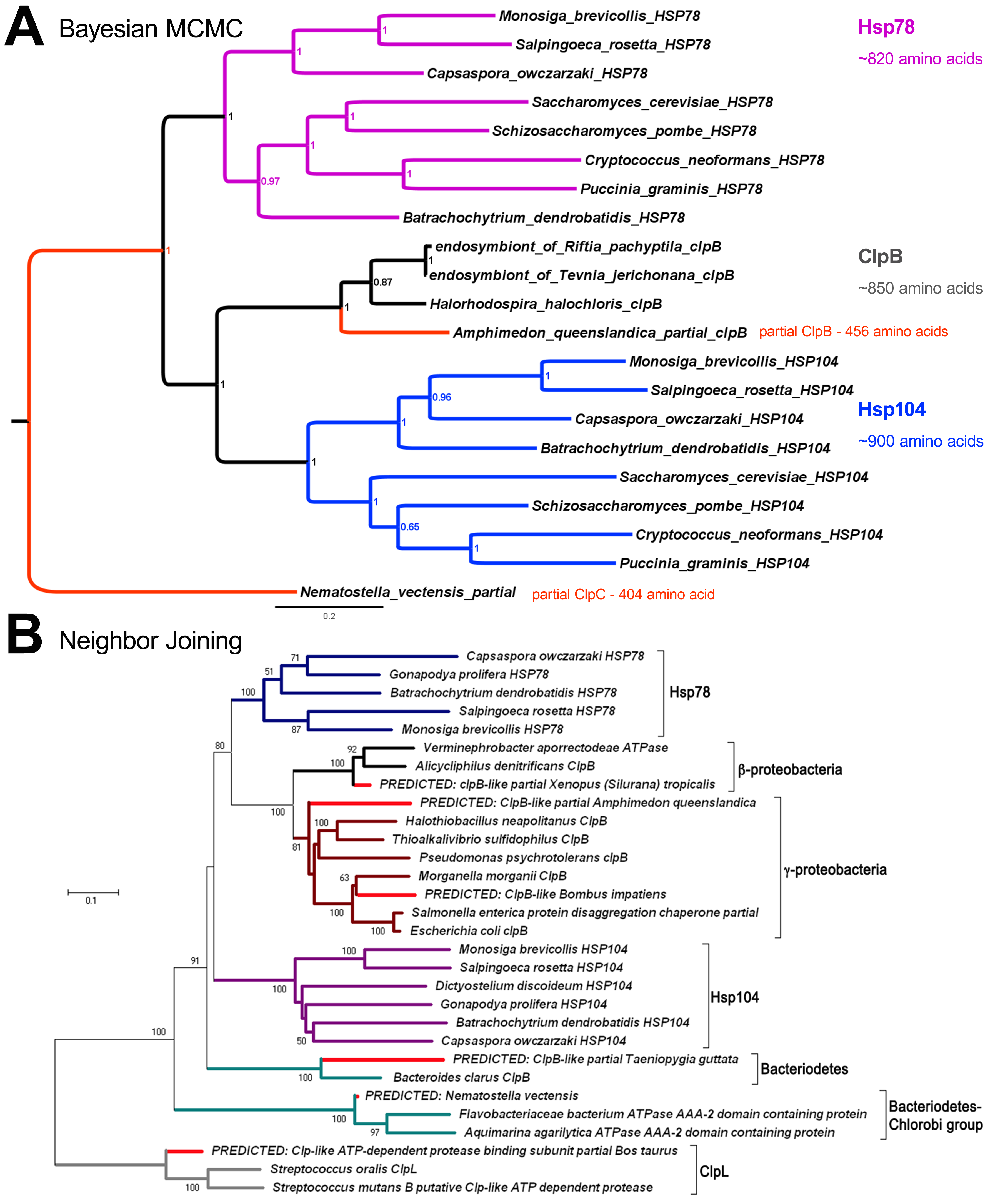

Supplement: S1 Fig — (A) Phylogenetic inference using Bayesian MCMC method, similar to Fig. 2A except a possible ortholog from the cnidarian N. vectensis is also included. This analysis shows that this sequence is correctly identified as a partial fragment of a clpC sequence, i.e., not a clpB gene. Topological convergence was achieved after 300,000 generations with a relative burn-in phase of 25% (MrBayes) [80–82]. Mixed models were tested, but WAG was the sole model favored upon completion. Average standard deviation of split frequencies was < 0.002. The tree was rooted between the entire clpB clade and the single clpC fragment. (B) The M. brevicollis Hsp104 sequence was used as a BLASTP query against the NCBI RefSeq database taxonomically restricted to animals. The highest scoring eight database hits were incorporated into the clpB phylogeny. High scoring bacterial sequences were also added to the phylogeny based on BLASTP analysis of each animal query against the RefSeq database taxonomically restricted to bacteria. Five of the top animal hits are shown in this tree. All positions with less than 50% site coverage were eliminated. There were a total of 862 positions in the final dataset. Bootstrap values lower than 50 are not shown. Animal sequences included in the tree are XP_002944514.1, Xenopus tropicalis; XP_003391862.1, Amphimedon queenslandica; XP_003493100.1, Bombus impatiens; XP_002199578.2, Taeniopygla guttata; gb EDO25580.1, Nematostella vectensis; and gb AAA68910.1, Bos taurus. (TIF) [file pone.0117192.s001.tif]

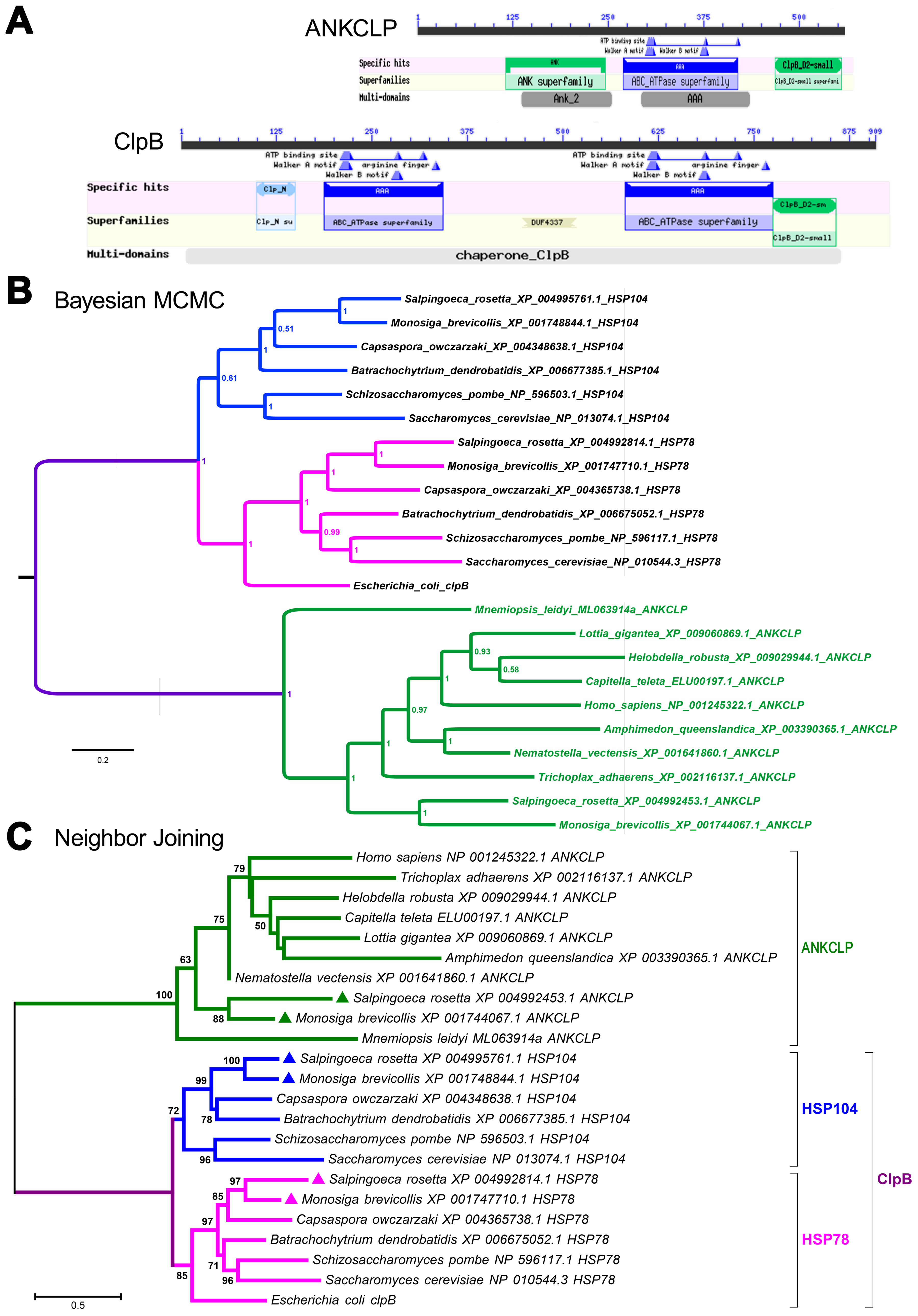

Supplement: S2 Fig — (A) Comparison of the ankyrin (ANK)–partial bacterial ClpB moiety to the actual ClpB domain structure, which contains two AAA+ domains. (B) Phylogenetic analysis by Bayesian MCMC (2,000,000 generations with average standard deviation of split frequencies < 0.0034 and with mixed amino acid models tested, 25% burn-in, and with WAG model having all posterior probability) showing that ANKCLP is found in holozoans and does not correspond to either bacterial clpB (E. coli) nor its eukaryotic orthologs HSP78 and HSP104. In the absence of HSP78 and HSP104 in animals, ANKCLP has been incorrectly annotated as “Casein lytic protease B, clpB”. Posterior probabilities shown at nodes. (C) Neighbor-Joining analysis of the same sequence alignment used in (B) with support from 500 bootstrap replicates. (TIF) [file pone.0117192.s002.tif]

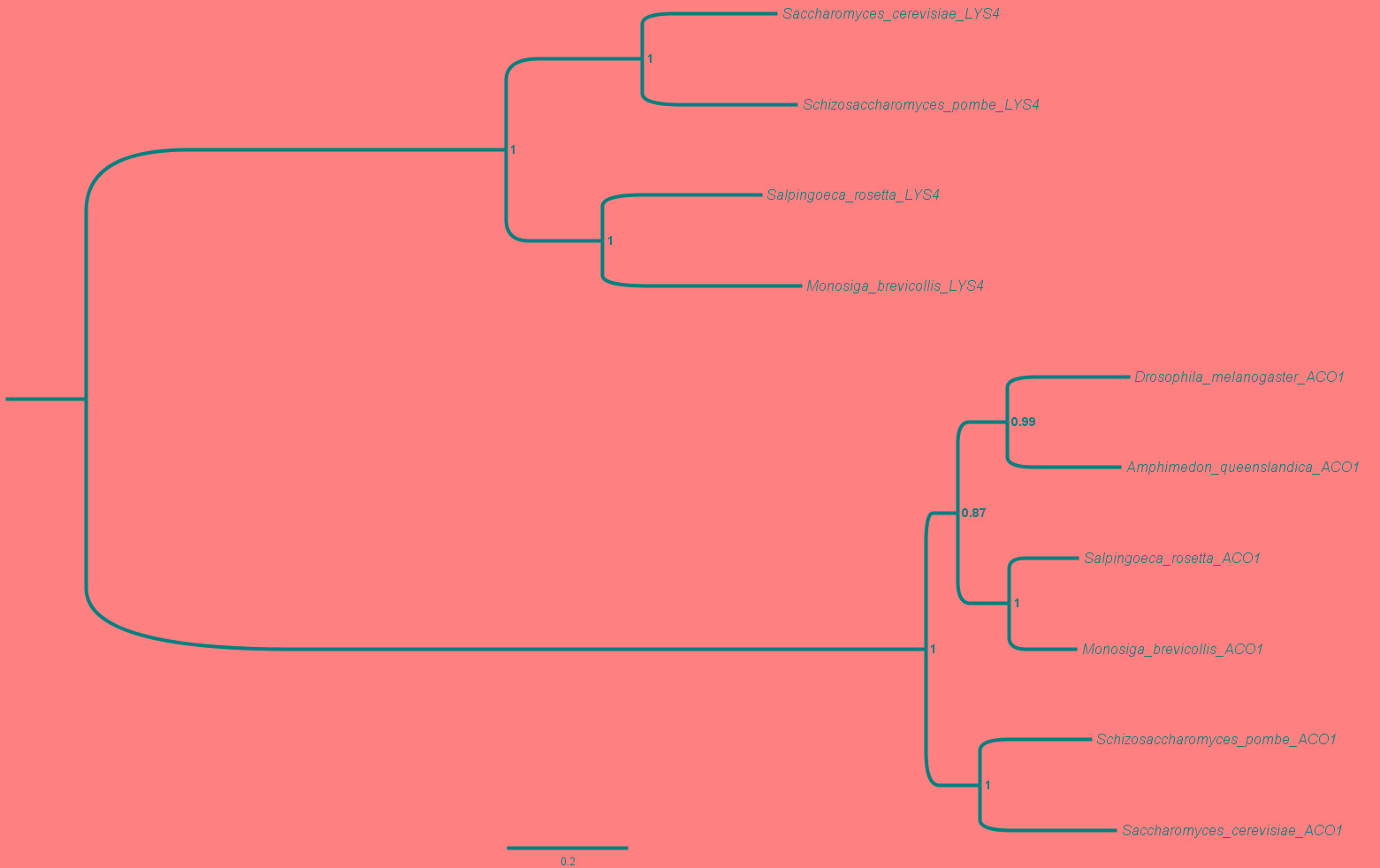

Supplement: S2 File — (ZIP) [file pone.0117192.s004.zip › M1_LYS4/LYS4_tree.jpg]
