# Supplementary material for: Metabolic and Chaperone Gene Loss Marks the Origin of Animals: Evidence for Hsp104 and Hsp78 Chaperones Sharing Mitochondrial Enzymes as Clients
Source: PLoS One. 2015 Feb 24;10(2):e0117192. doi: 10.1371/journal.pone.0117192 (PMC4339202; doi:10.1371/journal.pone.0117192)
Supplement: S3 File — Table A, Yeast strains used in this study; Table B, Primer sequences used in this study. (PDF) [file pone.0117192.s005.pdf]

Chaperone-Client Gene Loss in Origin of Animals Erives & Fassler (2014)

S3 File

Table A. Yeast strains used in this study

Strain Designation	Relevant Genotype	Origin
BY4730	*MATA leu2Δ0 met15Δ0 ura3Δ0*	
BY4700	*MATA ura3Δ0*	
BY4738	*MATα ura3Δ0 trp1Δ63*	
BY4742 *hsp78Δ*	*MATα hsp78ΔkanMX leu2Δ0 lys2Δ0 ura3Δ0 his3Δ1*	Winzeler et al., 1999
BY4742-*hsp104Δ*	*MATα hsp104ΔkanMX leu2Δ0 lys2Δ0 ura3Δ0 his3Δ1*	Winzeler et al., 1999
JF2478	*MATA hsp78ΔkanMX met15Δ0 his3Δ1 leu2Δ0 ura3Δ0*	This study
JF2479	*MATA hsp78ΔkanMX met15Δ0 his3Δ1 leu2Δ0 ura3Δ0*	This study
JF2480	*MATA hsp78ΔkanMX met15Δ0 his3Δ1 leu2Δ0 ura3Δ0*	This study
JF2473	*MATA hsp104ΔkanMX met15Δ0 leu2Δ0 ura3Δ0*	This study
JF2474	*MATA hsp104ΔkanMX met15Δ0 leu2Δ0 ura3Δ0*	This study
JF2498	*MATA hsp104ΔkanMX leu2Δ0 ura3Δ0*	This study
JF2494	*MATα hsp78ΔkanMX hsp104ΔkanMX his3Δ1 leu2Δ0 ura3Δ0*	This study
JF2495	*MATα hsp78ΔkanMX hsp104ΔkanMX his3Δ1 leu2Δ0 ura3Δ0*	This study
JF2516	*MATA hsp78ΔkanMX hsp104ΔkanMX met15Δ0 his3Δ1 leu2Δ0 ura3Δ0*	This study

Table B. Primer sequences used in this study.

Primer Designation	Sequence
Hsp104 -464F	5’ CCTTCTGCACCATTTTTAGAAAAA 3’
Hsp78 -393F	5’ ACGTAATACAATAAATAGGGAGCAGA 3’
kanB 250R	5’ CTGCAGCGAGGAGCCGTAAT 3’
